# Depth of interaction determination in monolithic scintillator with double side SiPM readout

**DOI:** 10.1186/s40658-017-0180-9

**Published:** 2017-02-16

**Authors:** Matteo Morrocchi, Giovanni Ambrosi, Maria Giuseppina Bisogni, Filippo Bosi, Marco Boretto, Piergiorgio Cerello, Maria Ionica, Ben Liu, Francesco Pennazio, Maria Antonietta Piliero, Giovanni Pirrone, Vasile Postolache, Richard Wheadon, Alberto Del Guerra

**Affiliations:** 1 University of Pisa and INFN, Sezione di Pisa, Italy; 2 INFN, Sezione di Perugia, Italy; 3 University of Torino and INFN, Sezione di Torino, Italy; 4 INFN, Sezione di Torino, Italy

**Keywords:** Monolithic scintillator, Depth of interaction, Positron emission tomography, Silicon photomultiplier

## Abstract

**Background:**

Monolithic scintillators read out by arrays of photodetectors represent a promising solution to obtain high spatial resolution and the depth of interaction (DOI) of the annihilation photon. We have recently investigated a detector geometry composed of a monolithic scintillator readout on two sides by silicon photomultiplier (SiPM) arrays, and we have proposed two parameters for the DOI determination: the difference in the number of triggered SiPMs on the two sides of the detector and the difference in the maximum collected signal on a single SiPM on each side. This work is focused on the DOI calibration and on the determination of the capability of our detector. For the DOI calibration, we studied a method which can be implemented also in detectors mounted in a full PET scanner. We used a PET detector module composed of a monolithic 20 × 20 × 10 mm^3^ LYSO scintillator crystal coupled on two opposite faces to two arrays of SiPMs. On each side, the scintillator was coupled to 6 × 6 SiPMs. In this paper, the two parameters previously proposed for the DOI determination were calibrated with two different methods. The first used a lateral scan of the detector with a collimated 511 keV pencil beam at steps of 0.5 mm to study the detector DOI capability, while the second used the background radiation of the ^176^Lu in the scintillator. The DOI determination capability was tested on different regions of the detector using each parameter and the combination of the two.

**Results:**

With both parameters for the DOI determination, in the lateral scan, the bias between the mean reconstructed DOI and the real beam position was lower than 0.3 mm, and the DOI distribution had a standard deviation of about 1.5 mm. When using the calibration with the radioactivity of the LYSO, the mean bias increased of about 0.2 mm but with no degradation of the standard deviation of the DOI distribution.

**Conclusions:**

The two parameters allow to achieve a DOI resolution comparable with the state of the art, giving a continuous information about the three-dimensional interaction position of the scintillation. These results were obtained by using simple estimators and a detector scalable to a whole PET system. The DOI calibration obtained using lutetium natural radioactivity gives results comparable to the other standard method but appears more readily applicable to detectors mounted in a full PET scanner.

## Background

One of the main contributions to the degradation of the spatial resolution in positron emission tomography scanners is represented by the parallax error [1]. This error comes from the fact that, when the 511 keV annihilation photons interact in the scintillators, the whole volume of the scintillators is taken into account to project the line of response (LOR) subtended by the two coincidence photons. The parallax error is negligible at the center of the field of view (FOV), while it increases at the periphery of the FOV. The correction of this effect is particularly important when the activity is distributed also at the periphery of the FOV, close to the detector ring. This happens for example in preclinical applications, in hybrid PET-MRI systems or in PET scanners for brain investigations.

The effect of the parallax error can be mitigated by introducing in the reconstruction algorithm the information about the depth of interaction of the photon in the scintillator, thus reducing the width of the LOR [2]. Simulations performed on a preclinical PET scanner featuring detectors with depth of interaction (DOI) capability [3] have shown that the diameter of the bore can be reduced down to 6 cm, increasing the overall scanner sensitivity. If a DOI resolution of about 4 times the pitch of the scintillator is reached, a uniform resolution in the whole region of interest (i.e., about 3 cm in diameter for mice) can be obtained. Since detectors for preclinical PET have usually spatial resolution close or better than 1 mm, this can be achieved when the DOI resolution is approximately 2 to 4 mm full width at half maximum (FWHM).

Furthermore, in applications where the scanner has a limited angle coverage or is composed of 2 or more planar heads, the adoption of the DOI information is essential for a more uniform sampling of the sinogram. Positron emission mammography (PEM), usually composed of two detector planes close to the region of interest, is an example of this kind of scanner architecture. In this application, high spatial resolution is required for the early detection of breast cancer tumor and the DOI is necessary to recover the resolution along the direction of the detector axis (which identifies the distance from the two detector planes). To fulfill the requirements of spatial resolution and uniformity, [4, 5] suggested that a DOI resolution between 2 and 3 mm is necessary.

Several solutions have been proposed to develop a PET detector with depth of interaction capability using pixelated crystals. Staggered layers of scintillator arrays [6] or of piled arrays of scintillator with different pulse shape (phoswich detector [7]) can be used to obtain the DOI information with a single side readout with a photomultiplier tube (PMT). Multiple layers of scintillators can be also identified adopting a light sharing scheme and observing the width of the light distribution [8]. In addition, it has been recently shown that a fluorescence coating can be used to tune the decay time of the detected light on a side of a pixelated crystal as a function of the DOI [9].

Silicon photodetectors have played a key role in this development, thanks to their high granularity and to the negligible reduction of the 511 keV photons flux through the silicon layer that composes the detector [10]. This last feature allows to place the photodetector between the radiation source and the scintillating crystal with a negligible absorption of the 511 keV photons. With the adoption of these photodetectors, multiple layers of detectors can be stacked to have an intrinsic DOI determination [11]. Furthermore, it has been shown that with a double side readout of a pixelated scintillator array the DOI can be determined by comparing the magnitude of the signal collected at the two sides and that a resolution close to 2 mm can be reached using this method [12–14]. The double side readout allows also to improve the time of flight capability of the detector due to the possibility to correct the difference in the length of the optical light path between the scintillation point and the photodetector [15].

An attractive alternative to pixelated crystals for high spatial resolution PET detectors is represented by monolithic scintillators coupled to arrays of photodetectors. The light sharing in an array of photodetectors allows the three-dimensional reconstruction of the scintillation event, with a high spatial resolution and depth of interaction positioning. By using monolithic crystals coupled to position sensitive photomultiplier tubes [16], to avalanche photodiodes [17] or to silicon photomultipliers (SiPM) [18–20], a spatial resolution close to the millimeter can be reached, and the depth of interaction can be reconstructed. Monolithic crystals coupled to a SiPM array can also reach the time resolution needed for time of flight applications [21].

The main disadvantages of large monolithic LYSO scintillator crystals are the high cost and the degradation of the spatial performances close to the edges of the detector. Several methods to reconstruct the position of the scintillation event have been developed. Basically, they make use of a set of look-up tables to describe the light distribution on the photodetectors as a function of the scintillation position [22]. Other methods are based on the comparison of the collected signal with an expected light shape [23], on the adoption of a center of gravity method, using specific algorithms to correct the artifacts at the edges of the scintillator [24], or on the optical coupling of neighbor crystals [25, 26].

The methods for the DOI reconstruction in monolithic scintillator crystals available in literature are based either on the clustering of the events collected in a frontal irradiation of the detector, or on the shape of the light distribution [27], or on the training of neural networks for spatial reconstruction [28, 29]. The reconstruction of the DOI information is usually based on large look-up tables containing the mean signals of the whole array of photosensors for a set of calibration position, thus requiring a long computational time and big amount of information to be transferred from the front end to the system that manages the data processing.

Therefore, the DOI estimation with monolithic crystals still requires improvements to provide a compromise between computational cost and DOI resolution. To this aim, we already demonstrated with a first prototype [30] a simple method for the DOI reconstruction in a monolithic scintillator with a double side readout. This method is based on the comparison of the signals on the two sides and requires a single one-dimensional look-up table (LUT), thus reducing the amount of data to be collected and transferred.

In this paper, we present the DOI performance of an improved PET detector module composed of a monolithic LYSO scintillator coupled on two opposite faces to two SiPM arrays developed in the framework of the 4DM-PET INFN project [31, 32]. We propose a new DOI calibration method which uses the natural lutetium radioactive background to determine the DOI value from the parameters we have defined for its estimation. The signals of the SiPMs on each side were processed by a 64-channel ASIC managed by a central processor that recorded the time stamp of the events and the energy collected in each pixel. Two methods were used to reconstruct the DOI. In the first one, the number of triggered SiPMs on each side of the detector was compared, while in the second, the maximum signal collected on each side on a single SiPM of the array was used. The combination of the two methods was also analyzed. The investigated parameters were calibrated by means of a lateral scan of the detector and using the natural radioactivity of the scintillator. Finally, the DOI capability was tested in different regions of the crystal.

## Methods

### Detector module

The aim of the 4DM-PET INFN collaboration is the development and the characterization of a PET detector composed of a monolithic scintillator coupled to arrays of SiPMs. To better exploit the spatial performance and the DOI capability of the detector, the scintillator is read out on two opposite sides by a high-granularity photodetector array. A first detector was developed, and we investigated its performance in terms of DOI resolution.

**Fig. 1:**
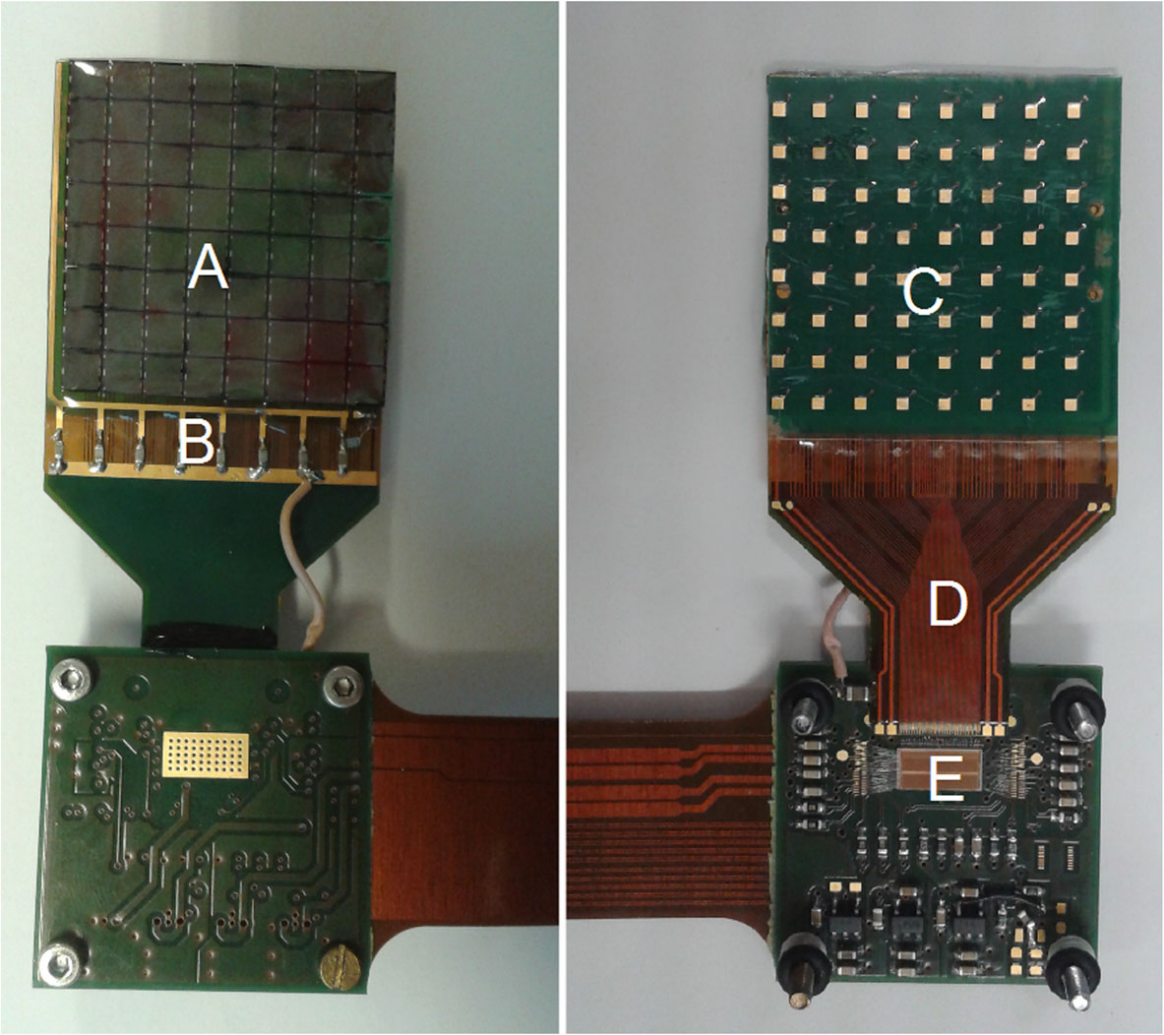
Picture of the SiPM matrix coupled to the PCB containing the ASIC. (*left*) The SiPM array (**a**) receives the bias from the pads on the top side through a bonding chain connected to the bias in the PCB (**b**). (*right*) On the back side, each channel is connected to a pad (**c**) used to probe the signal during the functionality test, and is collected in a fan-out (**d**). A flex cable connects the array to the ASIC (**e**)

The detector is composed of a 20 × 20 × 10 mm^3^ monolithic LYSO scintillator coupled on the entrance and exit surfaces to two arrays of SiPMs by means of silicon optical glue. The lateral sides of the crystal were black painted to collect only direct light while the faces coupled with the SiPM arrays were polished. A picture of the array of SiPMs and the front-end electronics is shown in Fig. 1. Each array (A in figure) was composed of 8 × 8 RGB SiPMs [33], produced by AdvanSiD, with a 3 × 3 mm^2^ active area and a 50 × 50 μm^2^ micro-cell size. Due to the size of the scintillator, only the central 6 × 6 subset was coupled to the crystal and connected to the readout system. Each array was custom developed and assembled, with a pitch of 3.6 × 3.6 mm^2^, corresponding to a packing factor of about 69%. The arrays were covered by an epoxy layer. To reduce the gap between adjacent pixels, the SiPMs were read from the bottom side and the bias was given to a column of eight SiPMs through a bonding chain on the SiPM pads (B). With this method, no extra dead space for routing is necessary on the sensitive surface. No degradation in the timing and in the signal shape was observed on single devices adopting this readout method. In the back side of the PCB containing the SiPMs, each channel was connected to a pad (C) used to probe the signal during the functionality test. A flex cable collected the signals in a fan-in (D) and connected the PCB hosting the ASIC to the SiPM array. The length and thickness of the flex allowed to rotate the matrix by 180° with respect to the ASIC to have a compact detection system. The detector is two sides buttable and can be used to assemble four detection modules in a 2 × 2 configuration with uniform pitch. In its final configuration, the detector will have the ASIC positioned over the SiPM array to avoid dead space between neighbor arrays. The thickness of the SiPM is few hundreds of micrometers [34] corresponding to an attenuation of about 1% of 511 keV incident photons (http://physics.nist.gov/PhysRefData/XrayMassCoef/ElemTab/z14.html). Considering also the thickness of the ASIC and of the PCB hosting ASIC and SiPMs, an overall attenuation between 3 and 5% can be expected for the 511 keV photon flux impinging the detector. Furthermore, no significant changes in performance of SiPMs have been observed for exposure to gamma radiation in signals composed of hundreds of photons [35, 36].

Each array was read out by the 64-channel ASIC TOFPET, developed within the ENDO-TOFPET collaboration [37, 38] and managed by an FPGA communicating with the PC. The working principle of the ASIC is summarized here. The input signal of the ASIC channel is amplified and sent to two discriminators. The first discriminator, with a low threshold, triggers a first time-to-digital-converter (TDC) that generates the information about the time stamp of the event. The second threshold, higher than the previous one, is used to validate the events. If, after triggering the first threshold, in a validation time window (set in the chip), the signal triggers also the higher threshold; the information about the time stamps and the energy is processed and stored; otherwise, the event is rejected. Data are then transmitted through a serial LVDS interface. The energy information is evaluated with the time-over-threshold (TOT) method, i.e, the time at which the signal returns below the higher threshold is recorded. Subtracting this information from the triggering time, we get the width of the signal. The width depends on the amount of detected light, hence on the energy released in the crystal. The TOT dependence on the energy is not linear and a calibration method is implemented in the ASIC.

The chip configuration allows to set the baseline of each channel (finely tuning the bias voltage of each sensor) and the trigger levels of the lower and higher thresholds by means of digital-to-analog converters with an 8-bit precision. An acquisition interface was implemented that handles the TDCs calibration and the TOT linearization using test signals generated inside the ASIC.

### Experimental setup

**Fig. 2:**
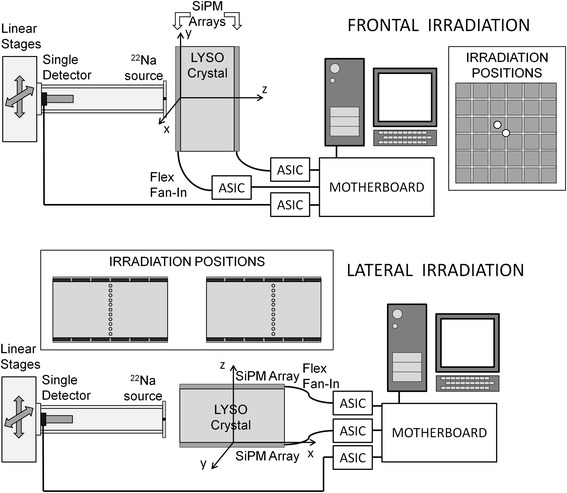
Schemes of the acquisition set-up for the frontal irradiation (*top*), and for the lateral irradiation (*bottom*) of the detector (not to scale). The reference axes and the irradiation position are reported in the two configurations

A single 3 × 3 mm^2^ SiPM coupled to a 4 × 4 × 20 mm^3^ LYSO scintillator crystal (single detector hereafter) was used in coincidence with the detector module to identify the two 511 keV annihilation photons of a ^22^Na radioactive source of about 1 mm diameter. The distance between the radioactive source and the entrance face of the coincidence detector was about 73 mm, while the distance between the source and the detector module was about 10 mm. The single detector was read out by the TOFPET ASIC, and the energy and timing information were recorded for each event. Every event detected by the module or by the single detector was acquired, and the coincidences were evaluated in the post-processing phase. A set of linear translators allowed to move the source and the single detector together in two directions, while the detector could be rotated to scan a lateral surface or the frontal surface of the crystal. No cooling was applied to the arrays, and all the acquisitions were made at a temperature of about 22 °C (not stabilized). A scheme of the acquisition setup is shown in Fig. 2 for both cases of frontal (top figure) and lateral irradiation (bottom figure). In the following, the axis will be named so to have the X and *Y* directions on the surface of the detector with the origin at the center of the detector, while the depth of interaction will be along the *Z* direction.

### Experimental measurements

Two acquisitions were performed by irradiating the frontal surface of the detector (Fig. 2—top), with the collimated beam at the center of the detector (*X* = 0 mm, *Y* = 0 mm) and at half the pitch of the SiPM arrays in both directions (*X* = −1.8 mm, *Y* = 1.8 mm). Since the centroid method was used to reconstruct the scintillation position, the *X*/*Y* spatial resolution close to the edges of the crystal has not been investigated yet, because edge effects degrade the resolution and introduce a bias in the results.

The detector was then rotated of 90° to scan a collimated beam onto the lateral surface of the detector along the *Y–Z* directions (Fig. 2—bottom). A scan at 0.5-mm steps along the *Z* direction was performed at the center of the lateral surface of the detector (*Y* = 0 mm) to investigate the depth of interaction capabilities. A second scan at *Y* = 1.8 mm (half of the SiPM pitch from the center) was also performed to check that the DOI performances were independent of the scintillation position. To discard Compton events, only an interval of ±4 mm around the nominal beam position was selected in the *Y* direction. The choice of the scan positions *Y* = 0 mm and *Y* = 1.8 mm was done to test the uniformity of DOI response by taking into account both the situations in which the scintillation occurs in a *X*-*Y* position corresponding to a sensitive area of the SiPM array or in an inactive region between two SiPMs. For each of the two *Y* positions, all the possible *X* positions across the crystal length [−10,10] mm were used, dividing the events according to the *X* position, as will be explained later. In this way, we could analyze the degradation of the DOI performance when one of the two coordinates is close to the crystal surface.

### Data processing

Data collected by each SiPM needed to be clustered to group together the signals belonging to the same scintillation event. To do so, the following steps were performed:

#### Event time separation

A clustering algorithm based on the time stamps was used. Given the first SiPM triggered on an array, when the time difference between two subsequent signals was lower than a cluster time window, the signals were marked as belonging to the same scintillation event. If the same channel was triggered more than once in the same scintillation event (i.e., the signal went over, then below and then again over the two thresholds) only the time stamp with the higher TOT was taken into account while all the TOTs were summed to obtain the energy information of that channel. Clustering was first performed on each side separately. Then the events on the two sides that were inside a coincidence window (considering the first trigger on each side) were marked as coincidence between the two sides. A time window of 50 ns was used both for the time clustering of the signals and for the evaluation of the coincidence between the two sides of the detector. We expect that the signal width should be greater than the decay time of the scintillator; therefore, the adoption of a narrower time window should not bring benefits in the event separation process.

#### Spatial clustering

For each data set belonging to the same scintillation event, as clustered in the previous step, an iterative region growing algorithm (here called cluster finding) was used to select the pixels to use in the elaboration. In each array, the algorithm starts from the pixel that has collected the maximum value of energy. The adjacent triggered pixels are assigned to the same event (cluster) if the measured signal is above a chosen threshold (the choice of the threshold is explained later in this section). The cluster is expanded until no new SiPMs are included. This method has many advantages. First, pixels that are triggered by noise far from the scintillation position can be rejected. Second, part of multiple interactions of the photon can be identified. At the end of the process, a cluster of neighbor SiPMs was obtained on each side of the detector. For each SiPM, the time stamp and the TOT were recorded and used to reconstruct the energy, position, and time.

#### Event filtering

To minimize the effect of multiple interactions and of noise, the interaction position was reconstructed separately on the two sides of the detector using the centroid method. The events in which the position on the two sides was farther than 5 mm were discarded. In our case, this corresponds to rejecting about 2.5% of the events. The number of triggered SiPMs was not fixed, but depended both on the position of the scintillation and on the energy released by the photon in the crystal. Only events that triggered at least one SiPM on each array and more than a total of three SiPMs were selected. The photopeak events were selected in the detector and in the single detector.

**Fig. 3:**
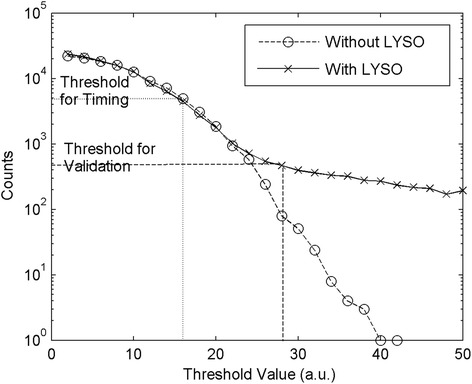
Number of triggers per second as a function of the validation threshold with (*crosses*) and without (*open circles*) the scintillator coupled to the SiPM. The threshold values to trigger the time stamp and to validate the events are shown (*dashed lines*)

The ASIC’s validation and timing thresholds were set independently for each channel in order to have a uniform behavior for all the SiPMs in the same array. Figure 3 shows the number of triggers as a function of the validation threshold for a single channel with and without the scintillator crystal, without any external radioactive source. The higher number of counts in the acquisition with the crystal (crosses) is due to the lutetium natural radioactivity. The lower threshold (dashed line in figure) was chosen so as to have a 5 kHz count rate (due to the SiPM dark noise more than to the crystal background). The validation threshold (dashed line in figure) was set to 12 DAC values higher than the previous one to discard signals generated by dark noise but to accept a high fraction of the signals generated by the LYSO background. The count rate at very low threshold (at the SiPM noise level) was affected by the ASIC dead time and did not correspond to the actual SiPM dark count rate. The remaining dark count events that were greater than the validation threshold and that triggered the channel readout independently from a scintillation event were discarded in the post processing by the cluster finding algorithm and by the rule that more than three SiPMs had to belong to each of the accepted events.

The threshold values selected with this method depend on the behavior of each SiPM (breakdown, gain, noise) and of each channel, but not on the number of counts due to the crystal, which depends on the position of each SiPM in the array. In fact, SiPMs close to the edges detect a lower amount of events due to the natural radioactivity of the LYSO with respect to the SiPMs at the center. The baselines on the ASICs were finely tuned to correct for the difference in the mean bias voltage between the two arrays, and they were set to have the same number of events above the validation threshold on both tiles (with the crystal alone and no other radioactive sources). A difference in the operating voltage of only 80 mV was found between the two arrays. A 2.5 V overvoltage was used. Even if this method for the threshold selection ensures uniformity in the data rate on both faces of the detector (and so, in principle on the threshold level), it is possible to have a residual difference in gain between the two arrays, and this difference can be corrected during the data analysis.

### Data analysis

#### Energy reconstruction

The signal collected on each triggered channel is proportional to the number of optical photons detected by the photodetector which is in turn related to the energy released in the scintillation event. Therefore, the energy was reconstructed by summing the signals belonging to the same cluster:

$$ E\propto {\displaystyle \sum }{E}_i+{\displaystyle \sum }{E}_j $$

where *i* runs on the SiPMs of the top matrix and *j* on the SiPMs of the bottom one, and *E*_*i(j)*_ are the energies collected by each SiPM on the two sides of the detector. In the analyzed configuration, due to the lateral black surfaces, only the photons in the solid angle subtended by the two arrays can be collected while the light impinging on the lateral absorbing surfaces is lost. Furthermore, only part of the two array surfaces is sensitive to the light, depending on the packing factor (i.e., the fraction of detector area covered by the SiPM) and on the fill factor of the single SiPM.

In the case of the lateral scan, the energy spectrum was evaluated separately for each analyzed region of the detector (described later in the “Timing” section), and the FWHM of the photopeak was then calculated and used for the energy filtering. The position of the energy peak depends both on the *X*-*Y* position and on the DOI because the fraction of collected scintillating light changes with the interaction position and because the SiPMs were calibrated to have a uniform trigger level, but a complete uniformity in gain is not ensured across the array.

The presence of events with a different number of involved SiPMs contributes to the uncertainty in the definition of the energy of the event. In fact, even if a very low threshold (corresponding to few photoelectrons) is set to trigger the readout of the SiPMs, the amount of the light that is not collected varies event by event. The adoption of a calibration method to ensure the uniformity in the trigger level of all the channels helps to reduce the variability in the collected energy, aligning the baseline of all the channels. In addition, the amount of collected energy is correlated to the total number of triggered SiPMs.

#### *X*-*Y* position

**Fig. 4:**
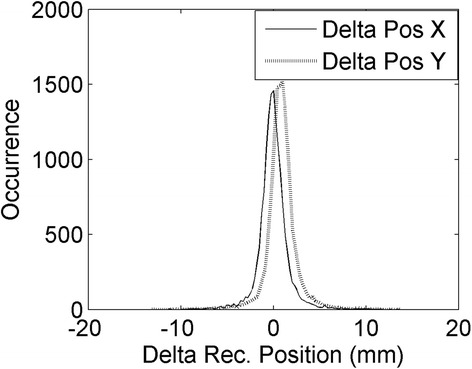
Difference in the reconstructed *X* positions (*continuous line*) and *Y* positions (*dotted line*) on the two faces of the crystal. Positions were reconstructed separately on the two sides using the center of gravity method on the SiPMs of the cluster

The centroid of the interaction position in the *X*-*Y* directions was first reconstructed separately on each SiPM array to verify that the two reconstructed points were close to each other and that the event could be accepted (see the “Data processing” section). Then the centroid was obtained using at the same time all the SiPMs in the clusters on both sides of the detector. To correct for the misalignment of the two tiles in the *X*-*Y* direction, a preliminary acquisition was performed using the ^176^Lu natural radioactivity. The position of the events was calculated by applying the centroid separately on each face of the detector, and the results for the two arrays were compared. The difference in the position on the two sides of the crystals is shown for the *X* and *Y* directions in Fig. 4; misalignments of Δ*Y* = 0.82 mm and Δ*X* = 0.21 mm were measured.

The measured misalignment was used to correct the reconstructed position of an event for the shift of the two arrays with the following equations:

$$ {X}_{\mathrm{Rec}}=\frac{{\displaystyle \sum }{X}_i{E}_i+{\displaystyle \sum}\left({X}_j-\varDelta X\right){E}_j}{{\displaystyle \sum }{E}_i+{\displaystyle \sum }{E}_j} $$

$$ {Y}_{\mathrm{Rec}}=\frac{{\displaystyle \sum }{Y}_i{E}_i+{\displaystyle \sum}\left({Y}_j-\varDelta Y\right){E}_j}{{\displaystyle \sum }{E}_i+{\displaystyle \sum }{E}_j} $$

where *i* and *j* are indices of the SiPMs of top and bottom arrays, respectively, and Δ*X* and Δ*Y* are the misalignments of the two arrays along the two directions. Only the translations between the two arrays on the *X* and *Y* direction were corrected, while a possible relative rotation was not taken into account.

#### Timing

In a preliminary calibration, the delay between the two ASICs was corrected. Since all the time stamps of the triggered SiPMs belonging to the same cluster in the two sides were recorded, all of them could be used to obtain the timing information about the cluster. In this work, only the first time stamp on each array was taken into account. Even if the choice of the first time stamp is not the optimal solution in terms of timing resolution (see for example [39]), this choice was good enough to select coincidence events in a time window of few nanoseconds. A coincidence time window of 8 ns was used here. The optimization of the timing performances of the setup is currently under study and is out of the scope of this paper, which is mainly focused on the depth of interaction reconstruction.

#### Depth of interaction

The DOI of the events was determined by comparing the signals collected on the two sides of the detector. For the DOI reconstruction, two different parameters were analyzed, hereafter called cluster size asymmetry and energy asymmetry. With a previous detector demonstrator, it has already been proved that both the parameters are able to reconstruct the DOI [30]. In this paper, also the result when combining the two methods was studied. For each of the two parameters a look-up table (LUT) was constructed to correlate the value of the parameter to the corresponding depth of interaction.

##### Cluster size asymmetry method

In this first method, the asymmetry between the number of pixels in the cluster (cluster size) in each tile was calculated using Eq. 1:

1 $$ {\mathrm{Asym}}_{\mathrm{CSIZE}}=\frac{{\mathrm{CSIZE}}_{\mathrm{TOP}}\hbox{-} {\mathrm{CSIZE}}_{\mathrm{BOTTOM}}}{{\mathrm{CSIZE}}_{\mathrm{TOP}}+{\mathrm{CSIZE}}_{\mathrm{BOTTOM}}} $$

where CSIZE_TOP_ and CSIZE_BOTTOM_ are the size of the clusters in the entrance and exit array. Only the pixels selected by the cluster finding algorithm were used to evaluate the depth of interaction. Due both to the critical angle at the epoxy/crystal interface and to the geometrical distribution of the optical photons on each pixel, clusters on the side closer to the scintillation position have a smaller number of pixels than the opposite one. Thus, this parameter is related to the DOI information, as shown in [30, 40].

##### Energy asymmetry method

The second method was based on the asymmetry of the energy deposited in the pixel with the higher signal amplitude in the entrance array (max_*i*_(*E*_*i*_)) and in the exit array (max_*i*_(*E*_*j*_)). The solid angle subtended by the active area of a single SiPM with respect to the scintillation position depends on the distance between the SiPM and the interaction point. We then expect that the maximum of the signal collected on the two sides is related to the DOI. The analyzed parameter is defined in Eq. 4:

2 $$ {\mathrm{Asym}}_{\mathrm{EMAX}}=\frac{{ \max}_i\left({E}_i\right)-{ \max}_j\left({E}_j\right)}{{ \max}_i\left({E}_i\right)+{ \max}_j\left({E}_j\right)} $$

Due to statistical fluctuations in the distribution of the emitted scintillation photons and to the misalignment between the two matrices, the maximum value measured on the two sides did not always correspond to SiPMs that faced each other; therefore, this condition was not imposed in the event selection. Using the energy asymmetry method, the variability in gain between the SiPMs was the main factor limiting the DOI resolution. A uniform gain among the SiPMs, together with a fine calibration of the ASIC thresholds is particularly important for the uniformity of this parameter in different regions of the detector.

##### Combined method

In this case, both parameters were used to evaluate the DOI. The DOI was found using the least square of the differences between the measured parameters and their expected values collected in two LUTs obtained in the calibration:

$$ \mathrm{D}\mathrm{O}\mathrm{I}=\underset{\mathrm{DOI}}{\mathrm{argmin}}\left({\left|{\mathrm{Asym}}_{\mathrm{EMAX}}-{\mathrm{LUT}}_{\mathrm{EMAX}}\left(\mathrm{DOI}\right)\right|}^2+{\left|{\mathrm{Asym}}_{\mathrm{CSIZE}}-{\mathrm{LUT}}_{\mathrm{CSIZE}}\left(\mathrm{DOI}\right)\right|}^2\right) $$

If the DOIs reconstructed using each of the two parameters differed more than 3 mm, the events were rejected.

**Fig. 5:**
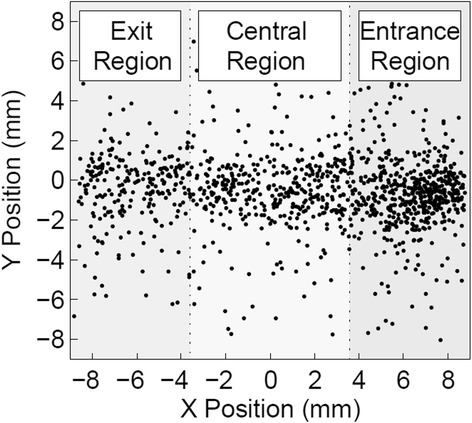
Example of the distribution of the events in the *X*-*Y* directions for a lateral irradiation at the center of the module. The three regions used for the analysis of the DOI determination capabilities are represented in figure

For each analyzed method, the mean bias of the reconstructed position $\left(\overline{\left|{Z}_{\mathrm{real}}-\overline{Z_{\mathrm{rec}}}\right|}\right)$ was analyzed for the whole *Z* range and for the *Z* interval [2 mm, 8 mm], together with the mean value of the standard deviation of the reconstructed distribution (*σ*). To compare the DOI capability at the center of the detector and off-center, the results obtained with the combined method were also analyzed dividing the detector in three different regions, according to the reconstructed *X* position: an “entrance region”, close to the entrance surface of the collimated beam, a “central region”, subtended by the two central rows of SiPMs, and an “exit region”, on the opposite side of the detector. The three regions are shown in Fig. 5, superimposed on the *X*-*Y* spatial distribution of a representative subset of the events in a scan.

The effect of saturation of the SiPMs given by scintillating events close to the matrix surface was taken into account during the calibration of the LUT used for the DOI estimation. Therefore, it is expected that no bias is introduced in the obtained DOI. Despite this, in the case of the energy asymmetry method, saturation of signals contributes to reduce the specificity of the parameter and a slight degradation of the resolution can be expected at the boundaries of the DOI range.

### DOI calibration

By definition, both the parameters introduced for the DOI reconstruction are bounded in the interval −1 < DOI < 1. The conversion of the asymmetry values to DOI values needs to be calibrated, and this can be done by means of a lateral scan of the detector or using physical assumptions on the distribution of the events in the volume of the crystal, as explained in the following:

#### DOI calibration with lateral scan

This calibration method could be difficult to implement in a whole scanner, but it is useful in the initial characterization of the detector to understand its performance. Data collected in the scan with the beam at the *Y* = 0 mm position were divided in two ensembles of the same size, called calibration set and test set. An energy filter around the photopeak and a spatial filter around the beam position were applied to both the calibration and the test sets. To take into account the different amounts of light collected at the center of the detector, a different energy filter was applied to the events subtending the central region of the detector (−3.6 mm < *X* < 3.6 mm) with respect to the edges.

In the calibration set, for each beam position, the values of energy asymmetry and of cluster size asymmetry parameters were obtained by fitting the peak of their distribution with a Gaussian curve. The mean of the fitted distributions was used for the calibration. Since the distribution of the parameters is highly asymmetric close to *Z* = 0 mm and *Z* = 10 mm, the tails of the distributions were not considered in the fit, and the asymmetry values corresponding to *Z* = 0 mm and *Z* = 10 mm were imposed to be equal to the two limits of the distributions (−1 and 1). The calibration values (i.e., the mean of the fitted distributions) obtained for each point of the lateral scan were then linearly interpolated to obtain, for each of the two asymmetry parameters, a LUT on a grid of 0.1 mm.

For each acquired event in the test set, the cluster size asymmetry and the energy asymmetry were evaluated. For each asymmetry parameter, the closer values in the LUT were searched and the corresponding DOI value was assigned to the event; thus, an independent value of DOI was obtained for each of the two methods. To test the consistency of the calibration in a different region of the detector, the calibration obtained with the scan at the center of the detector was applied also to the second scan performed at *Y* = 1.8 mm.

#### DOI calibration using the LYSO natural radioactivity

Several calibration methods have been proposed that are fast and easy to implement in a real system. Such methods are usually based on a scan of the module using a group of collimated beams, a fan beam irradiation, or a known event distribution in the module [24, 41, 42]. The natural radioactivity of the LYSO has been used to calibrate the DOI response in modules composed of pixellated scintillators with double side readout [43, 44], and the same method was applied here to calibrate the DOI parameters. This method is particularly advantageous because it allows to obtain the DOI calibration without the need of a dedicated measurement setup, and it can be applied to detectors mounted in a full PET scanner.

**Fig. 6:**
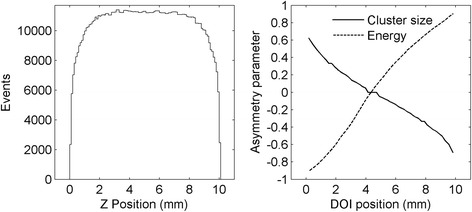
(*left*) Distribution of the centroid of the simulated background events in the *Z* direction. (*right*) Cluster size asymmetry (*solid line*) and maximum energy asymmetry (*dashed line*) calibration function obtained correlating the simulation with the acquisition of the ^176^Lu background

An acquisition of the background radiation of the lutetium in the scintillator was performed. Even if the distribution of the ^176^Lu in the scintillator is assumed to be uniform, the distribution of the detected events can differ from this, due to the path of the de-excitation photons before their eventual absorption and to the different sensitivity of the detector at different DOIs for low-energy events. Although this last effect can be partially mitigated introducing a software threshold on the energy of the event, in previous studies, it has been observed that the application of an energy window to the event selection used for the calibration does not significantly affect the results [44]. To take into account the path of the de-excitation photons, a Geant4/GAMOS Monte Carlo simulation was performed [45, 46]. The decay of the ^176^Lu was simulated for the 20 × 20 × 10 mm^3^ LYSO scintillator used experimentally, and the centroid of the beta and photons interactions that occurred for each decay in the *Z* direction was reconstructed. The distribution of the obtained depths is shown in Fig. 6-left. It can be seen that the assumption of a uniform distribution of the event centroids is no longer valid close to the two matrices (i.e., first and last 2 mm in DOI) due to the partial escape of the de-excitation photons from the scintillator.

The calibration LUTs for the cluster size asymmetry and for the energy asymmetry parameters were created by correlating the distributions of the two parameters obtained in the acquisition of the lutetium radioactivity with the expected depth of interaction predicted by the simulation. To do so, the distributions of the two parameters and of the simulated DOIs were divided in 50 percentiles to directly match, for each interval, the mean values of the DOI with the mean value of each parameter. The calibration curves are shown in Fig. 6-right: the solid line represents the dependency of the cluster size asymmetry on the DOI position obtained correlating the simulation with the acquisition of the ^176^Lu background, while the dashed line is related to the maximum energy asymmetry.

**Fig. 7:**
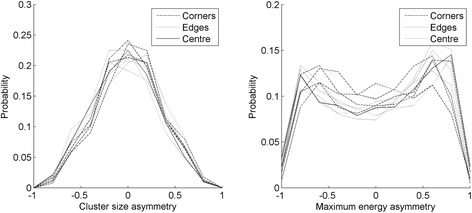
Distribution of the cluster size asymmetry parameter (*left*) and of the energy asymmetry parameter (*right*) for different regions of the detectors corresponding to the four edges, the four corners and the central region, obtained with the natural ^176^Lu background

All of the events occurred in the scintillator were used to reconstruct the LUTs despite their *X*-*Y* interaction position. To verify that the events that occurred in different regions of the detector behaved consistently, the distributions of the asymmetry parameters obtained with the background radioactivity were plotted separately for nine regions of the detector. Each region corresponded to a 2 × 2 group of adjacent SiPMs and identified different portions of the crystal: four regions corresponded to extreme values of both the *X* and *Y* coordinates (hereafter called corners), four regions had only one of the two coordinates at the boundary (called edges), and one region identified the center (called center). The distributions of the two DOI parameters for these nine regions are shown in Fig. 7. As it can be seen, the distributions of the cluster size asymmetry are quite consistent in the whole area of the detector, while the maximum asymmetry parameter distribution has a higher variability. This is partially due to the method implemented for the channels equalization, that is based more on the normalization of the trigger level than on the gains of the channels.

## Results

### Frontal irradiation

**Fig. 8:**
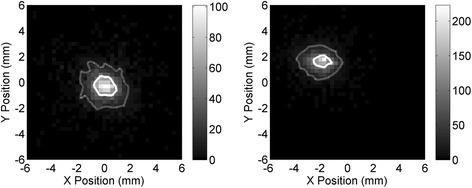
Spatial distributions obtained with the collimated beam at the center of the detector (*left*) and at 1.8 mm from the center in both directions (*right*), corresponding to the center of one of the SiPM of each matrix. Continuous lines represent the contour corresponding to the FWHM of the distribution while dotted lines correspond to the FWTM of the distribution

**Fig. 9:**
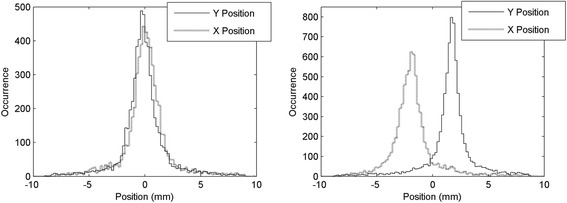
*X* distribution (*dotted lines*) and *Y* distribution (*solid line*) of the events obtained in the two acquisitions of Fig. 8, with the coincidence beam at the center of the crystal (*left*) and at 1.8 mm from the center in both directions (*right*)

The spatial distribution of the reconstructed events in the *X*-*Y* directions is shown in Fig. 8 for the two beam positions (left: *X* = 0 mm and *Y* = 0 mm, right: *X* = −1.8 mm and *Y* = 1.8 mm). The contours corresponding to the full width at half maximum (FWHM, continuous lines) and to the full width at tenth maximum (FWTM, dotted lines) of the distribution are also shown. The value of the width at half and at tenth maximum were obtained as the mean diameter of the contours, giving FWHM = 1.5 mm and FWTM = 3.6 mm for the distribution at the center and FWHM = 1.2 mm and FWTM = 3.0 mm at 1.8 mm from the center, without subtracting the contribution of the beam spot size. Figure 9 shows the distribution of the projection of the reconstructed positions in the two directions, with the beam at the center of the detector (left) and translated of half the pitch size in both directions (right), again without subtracting the beam spot size. The distance between the center of the two distributions (at the center and at half the pitch) is 1.79 mm in the *X* direction and 1.92 mm in the *Y* direction, close to the expected value of 1.8 mm (half the pitch). This suggests that the width of the distribution obtained at the center of the detector is not distorted by the effects of the centroid algorithm used here. The reconstructed positions and width of the distributions are summarized in Table 1.

**Table 1:** Reconstructed position, FWHM, and FWTM in the two beam positions with frontal irradiation of the detector

Beam position (mm)		*X*_Rec_ (mm)	*Y*_Rec_ (mm)	FWHM (mm)	FWTM (mm)
*X*	*Y*				
0.0	0.0	−0.18	−0.23	1.5	3.6
−1.8	1.8	−1.97	−1.69	1.2	3.0

**Fig. 10:**
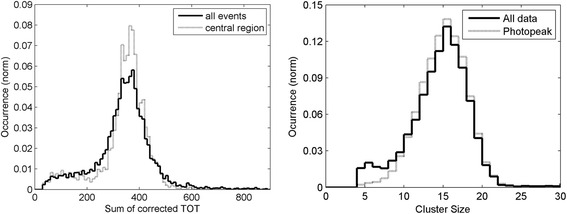
(*left*) Energy spectra with the collimated beam at the center of the detector using all the events (*continuous line*) and only the events inside the FWHM of the distribution (*dotted line*). (*right*) Total number of SiPMs in the clusters in the two sides of the detector for the whole data-set (*solid line*) and for the photopeak events selected in the energy window (*dotted line*)

The energy spectrum with the collimated beam at the center of the detector was analyzed considering the whole ensemble of events (Fig. 10-left, solid line) and only the events within one FWHM of the distribution (Fig. 10-left, dotted line). Since the energy collected in each channel was measured with the method of the time-over-threshold, the baseline of each channel differed from the baseline of the energy spectrum. An acquisition with several radioactive sources with different energies should be carried out to determine the position of the baseline in the energy spectrum and then to convert the spectrum from arbitrary units into kiloelectron volt.

The distribution of the total number of SiPMs in the clusters on the two sides of the detector is plotted in Fig. 10-right for the whole data set (solid line) and for the photopeak events selected in the energy window (dotted line). This distribution is peaked around 15 SiPMs; therefore, the requirement that more than three SiPMs have to be triggered in total on the two sides of the detector is compatible with the number of SiPMs actually triggered by a scintillation event.

### Lateral irradiation

**Fig. 11:**
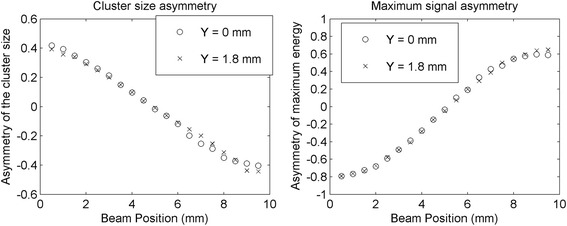
Cluster size asymmetry (*left*) and energy asymmetry (*right*) obtained in the lateral *Z* scans at the center of the lateral surfaces (*circles*) and at 1.8 mm from the center (*crosses*)

Figure 11 shows the mean value of the cluster size asymmetry (left) and of the energy asymmetry (right) parameters as a function of the actual beam position, for the two lateral *Z* scans (open dots refer to *Y* = 0 mm and crosses refer to *Y* = 1.8 mm). No significant differences in the trend of the mean are visible when changing the beam position. The energy asymmetry parameter shows a more evident non linearity in the first and last 2 mm with respect to the cluster size asymmetry parameter. The two asymmetry parameters have a monotonic dependence on the depth of interaction. It should be noted that the slope of the two variables as a function of the DOI is opposite. In fact, while the maximum collected signal increases closer to the SiPM, the number of SiPMs in the cluster increases farther from them.

**Fig. 12:**
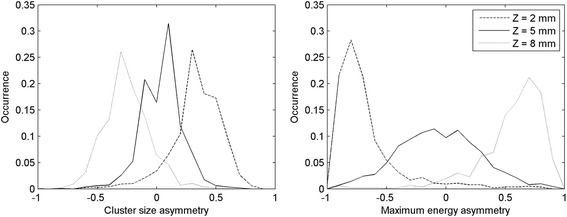
Distribution of the cluster size asymmetry (*left*) and of the maximum energy asymmetry (*right*) for the points [2 mm, 5 mm, 8 mm], in the scan at the center of the lateral surface

The profiles of the distributions of the cluster size asymmetry (left) and of the energy asymmetry (right) corresponding to the *Z* beam positions [2 mm, 5 mm, 8 mm] with the beam at the *Y* = 0 mm position are shown in Fig. 12.

### Results on DOI calibration

**Fig. 13:**
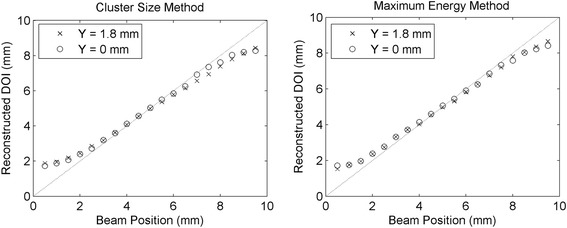
Reconstructed depth of interaction as a function of the collimated beam position (*Z* direction) using the cluster size method (*left*) and the maximum collected energy method (*right*). The line in the plots represents the bisector of the plane, corresponding to an ideal reconstruction of the beam position

**Fig. 14:**
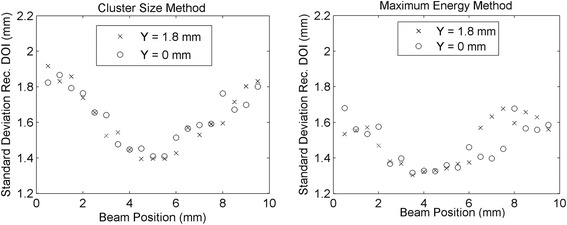
Standard deviation of the reconstructed depth of interaction using the cluster size method (*left*) and the maximum collected energy method (*right*)

**Fig. 15:**
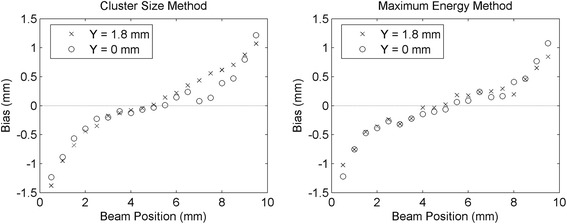
Bias of the mean reconstructed depth of interaction with respect to the real beam position, by using the cluster size method (*left*) and the maximum collected energy method (*right*)

**Fig. 16:**
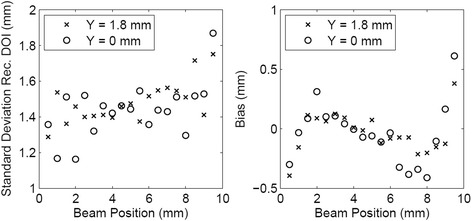
Standard deviation (*left*) and bias (*right*) of the reconstructed DOI distributions using the combination of the two asymmetry parameters and the calibration obtained with the lateral scan of the detector

The distributions of the two asymmetry parameters obtained in the two lateral *Z* scans were converted into depth of interaction values using the LUTs obtained as explained in section 2.6. The mean DOI reconstructed positions are shown in Fig. 13. In both *Z* scans, the mean standard deviation in each acquisition was σ = 1.5 mm using the maximum asymmetry method, and σ = 1.6 mm using the cluster size asymmetry method. The value of the standard deviation for each beam position for the two methods is shown in Fig. 14. The difference between the real beam position (*Z*_real_) and the mean reconstructed position (*Z*_rec_), hereafter called bias, is shown in Fig. 15. In the three figures circles refer to *Y* = 0 mm and crosses refer to *Y* = 1.8 mm. With both methods a mean bias lower than 0.3 mm was obtained in the interval *Z*_real_ = [2 mm, 8 mm], while the bias was higher in the first and last millimeters of the crystal. The results obtained with the combination of the two methods are shown in Fig. 16. A mean bias lower than 0.2 mm was obtained on both lateral *Z* scans with a mean standard deviation of the DOI distribution better than 1.5 mm. The rejection of events in which the two parameters provided two DOI values different more than 3 mm partially removed the contribution of double interactions in the scintillator, and between 2 and 4% of the events were rejected. Table 2 summarizes the results obtained in the two lateral *Z* scans of the detector.

**Table 2:** Summary of depth of interaction reconstruction capability of the detector tested in the two scans and of the overall results obtained using both scans together

*Y* beam position	Reconstruction method	Bias (mm) *Z*_real_ in [0 mm, 10 mm]	Bias (mm) *Z*_real_ in [2 mm, 8 mm]	σ (mm) *Z*_real_ in [0 mm, 10 mm]
0 mm	Cluster size	0.38	0.16	1.62
	Maximum	0.38	0.20	1.47
	Combined	0.19	0.18	1.44
1.8 mm	Cluster size	0.48	0.27	1.62
	Maximum	0.35	0.20	1.49
	Combined	0.13	0.09	1.48
All	Cluster size	0.42	0.22	1.62
	Maximum	0.37	0.21	1.48
	Combined	0.17	0.14	1.47

The mean bias and the standard deviation of the reconstructed distribution for the three regions are summarized in Table 3. The DOI performances are similar in the “entrance region” and in the “central region” of the detector, while an increase of the bias and of the standard deviation was observed in the “exit region”. The increase of the standard deviation could be partially due to the broadening of the collimated beam.

**Table 3:** Summary of depth of interaction determination in three different regions of the detector; the entrance and exit region represent portions of the detector close to the *X* boundaries while the central part is subtended by the two central SiPMs

*Y* beam position	Investigated region	Bias (mm) *Z*_real_ in [0 mm, 10 mm]	Bias (mm) *Z*_real_ in [2 mm, 8 mm]	σ (mm) *Z*_real_ in [0 mm, 10 mm]
0 mm	Entrance *X* > 3.6 mm	0.20	0.21	1.33
	Central region	0.21	0.18	1.54
	Exit *X* < −3.6 mm	0.25	0.21	1.60
1.8 mm	Entrance *X* > 3.6 mm	0.19	0.22	1.39
	Central Region	0.18	0.11	1.46
	Exit *X* < −3.6 mm	0.41	0.46	1.60

**Fig. 17:**
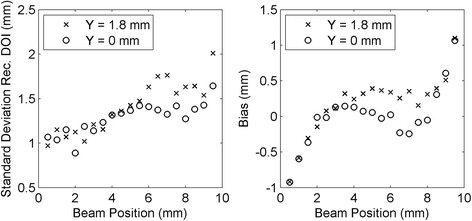
Standard deviation (*left*) and bias (*right*) of the reconstructed DOI distributions using the combination of the two asymmetry parameters and the calibration obtained with the ^176^Lu background

The results obtained with the calibration performed using the ^176^Lu background in terms of standard deviation of the reconstructed distribution (left) and bias (right) are shown in Fig. 17 and summarized in Table 4.

**Table 4:** Summary of depth of interaction reconstruction capability of the detector using the combination of the two investigated parameters, with both calibration methods: using the natural radioactivity of the lutetium and with the lateral scan

*Y* beam position	Calibration method	Bias (mm) *Z*_real_ in [0 mm, 10 mm]	Bias (mm) *Z*_real_ in [2 mm, 8 mm]	σ (mm) *Z*_real_ in [0 mm, 10 mm]
0 mm	Lutetium	0.27	0.09	1.28
	Lateral scan	0.19	0.18	1.44
1.8 mm	Lutetium	0.38	0.26	1.41
	Lateral scan	0.13	0.09	1.48

## Discussion

The high spatial resolution obtained at the center of the detector (Fig. 6), not corrected for the beam size, suggests that the 4DM-PET approach can be applied to preclinical PET detectors. For the position reconstruction, methods more effective than the centroid need to be considered because the results obtained with this method can still be affected by residual non-linearity after TOT correction. In fact, the centroid method usually shifts towards the center of the detector the *X*-*Y* positions that are actually close to the edges. Therefore, before determining the real FWHM of the spatial distribution achievable at the center, it is necessary to verify that this artifact is not present in the investigated area. With the frontal irradiation at 1.8 mm from the center of the detector, it was verified that the position of the center of the distribution was determined in the expected position, without any bias effect induced by the centroid method. This validated the value of FWHM obtained at the center of the detector. To fully determine the *X*/*Y* spatial resolution of the detector out of the center, a calibration of the response of the detector should be done, scanning the frontal surface of the detector on a regular grid.

Both the asymmetry parameters proposed for the DOI estimation depend on the *Z* position of the beam, as shown in Fig. 8, and therefore can be used for the DOI reconstruction. In addition, the two parameters are monotonic in the *Z* position, even if both show a non-linearity of the DOI close to the SiPM arrays, corresponding to a loss in accuracy in those regions of the detector. The values obtained in the lateral scan comparing the maximum collected signal on each side are not symmetric with respect to the half depth of the detector (i.e. the values shown in Fig. 8-right are confined in the asymmetric range [−0.8, 0.6]), conversely to the distribution of the calibration values used for the method of the cluster size asymmetry that is ranging in approximately [−0.4, 0.4]. Since the calibration was performed to get a uniform trigger level on both sides of the detector, we expected a symmetric distribution of the LUT related to the cluster size asymmetry, while the distributions of the maximum energy asymmetry was less controlled. Even if a software correction can be applied to the detected signals to obtain a fixed gain, this effect is already taken into account in the LUT determination and does not introduce artifacts in the DOI.

The DOI obtained with the “cluster size” method had a larger standard deviation with respect to the “maximum signal” method. At the edges of the crystal, an additional increase of the bias was visible, in agreement with what has already been observed in monolithic scintillator with single side readout by other groups (see e.g. [41]). This bias is due to the partial loss of a tail of the collimated beam, which shifts the mean reconstructed position towards the center of the crystal. Close to the edges, also the distribution of the double interaction events was not symmetric with respect to the axis of the beam because in the *Z* direction there is a higher fraction of the volume of scintillator toward the center of the detector than in the other direction. Even if part of the events that include a double interaction in the scintillator is rejected by the cluster finding algorithm, the discrimination does not work if the path between the two scintillations is too short; thus a fraction of these events contributes to the obtained distribution. Although the bias is also present in the distribution used for the generation of the LUTs for the DOI reconstruction (see Fig. 8), these effects are mitigated in the calibration because the tails of the distributions were not taken into account and the calibration value was evaluated only around the peak of the distribution. For a more accurate result, separate calibrations could be performed in sub-regions of the detector to take into account edge effects and local non-uniformities in sensitivity, optical coupling, and gain.

The DOI estimation obtained combining the two parameters provided comparable results in terms of standard deviation of the distributions and an improvement in terms of bias at the entrance and exit region of the collimated beam in the detector. This improvement in the combined method is probably due to the rejection of part of the events that interacted more than once in the scintillator and that provided DOI values very different from the real beam position, and that were not identified when only one parameter was used. Furthermore, the use of more than one parameter in the DOI estimation can ensure a higher accuracy. The calibration implemented using the background radioactivity of the ^176^Lu applied to the DOI identification using the combined method exhibits comparable results in terms of both mean bias and standard deviation, even if the bias in the entrance and exit regions is larger than that obtained with the lateral scan calibration. Furthermore, in this case, the difference in performances between the two scans is greater, suggesting that some benefits could arise from a set of calibrations specific for separated regions of the detector.

With respect to our previous study [28], the new readout system can be triggered by lower signals, thus sampling the light distribution in a larger cluster of SiPMs. The SiPM arrays adopted here have been optimized to reduce the inactive spaces between SiPMs. In addition, the architecture of the detector allows to place the detectors side by side, without dead spaces. Finally, in this study, a LUT has been created for each parameter to determine the DOI. This method allows to finely model the asymmetry parameter for each DOI, and it can be calibrated using the natural background of the scintillator.

The DOI estimation methods proposed here do not require the readout of the whole light distribution on the entire detector and exhibit an efficient identification of the DOI because they are based on a single one-dimensional LUT. In fact, in this paper, it has been shown that the DOI determination can be simplified because the same DOI calibration can be used both at the center and at the edges of the detector. The adoption of the combined parameter reduces the computational efficiency because two sets of LUTs need to be used and the results obtained separately with the two methods need to be compared. However, the combined method exhibits a lower mean bias. The easiness of the proposed methods allows the implementation of the DOI reconstruction directly in the readout electronics (i.e., on FPGA), thus reducing the complexity of the acquisition system and of the data elaboration process and making this solution suitable for application in a whole PET scanner. Even if the DOI resolution obtained in this work is close to state of the art results obtained with monolithic crystals [29, 41], a comparison with the results obtained by other groups using thicker scintillators, e.g., [47], is not straightforward due to the different signal-to-noise ratio achievable on the SiPMs with a different dimension of the scintillator.

The treatment of the lateral faces with an absorbing black paint has advantages and disadvantages. With respect to the two parameters investigated here for the DOI identification, the adoption of black surfaces may reduce the dependence of the maximum energy asymmetry parameter on the *X*/*Y* position in the crystal because only the direct light is collected, while it is difficult to estimate the effect of a reflective surface on the uniformity of the cluster size asymmetry close to the edges. In terms of spatial resolution, both absorbing [48] and reflective surfaces [18] have been proposed for monolithic scintillators with excellent results in the event positioning. The main difference between these two approaches is in the light collection efficiency, and the adoption of reflective surfaces might be particularly useful with thick monolithic scintillators, in which the direct light subtends a limited solid angle [49].

## Conclusions

A PET detector composed of a 20 × 20 × 10 mm^3^ monolithic LYSO scintillator crystal coupled on the entrance and exit surface to a 6 × 6 SiPMs matrix was developed and its capability to determine the depth of interaction was investigated. The depth of interaction was reconstructed comparing the signal collected on the two tiles, by means of two parameters: the asymmetry of the maximum energy collected on a single SiPM on the two sides and the asymmetry of the number of pixels triggered on the two sides. The two variables were first calibrated using a scan of the lateral face of the detector at a regular pitch of 0.5 mm and at the center of the lateral side (*Y* = 0 mm). The DOI capabilities and the calibration method were then tested using a test set in the same positions of the calibration scan and a second set at half the SiPM pitch away from the center of the lateral side. For the depth of interaction profiles on each scan position, we obtained a mean standard deviation of σ = 1.5 mm using the maximum asymmetry method and of σ = 1.6 mm using the cluster size asymmetry method. The mean bias of the reconstructed depth of interaction was lower than 0.3 mm in the *Z* interval [2 mm, 8 mm], while the bias and the standard deviation of the DOI distribution were higher close to the two faces of the crystal. This effect is partially due to Compton events that were not discarded in the energy and spatial selection. A smaller bias was obtained combining the two parameters for the DOI estimation. It was also demonstrated that the parameters can be calibrated using the natural radioactivity of the LYSO, with an increase of the mean bias of about 0.2 mm and with no degradation of the standard deviation of the DOI distribution.

It was demonstrated that the DOI can be determined by comparing the signals collected at the two sides of a monolithic scintillator crystal. This allows to achieve a DOI resolution comparable with the state of the art, with an easy reconstruction method and one-dimensional LUTs. The DOI determination capability, together with the high spatial resolution at the center of the detector, makes this detector suitable for high spatial resolution applications like preclinical PET or dedicated PET detectors. In addition, we proposed and tested a DOI calibration method which exploits Lutetium natural radioactivity and could easily be applied also to full PET systems.
